# The Systematic Bias of Ingestible Core Temperature Sensors Requires a Correction by Linear Regression

**DOI:** 10.3389/fphys.2017.00260

**Published:** 2017-04-27

**Authors:** Andrew P. Hunt, Aaron J. E. Bach, David N. Borg, Joseph T. Costello, Ian B. Stewart

**Affiliations:** ^1^Faculty of Health, School of Exercise and Nutrition Sciences, Institute of Health and Biomedical Innovation, Queensland University of TechnologyBrisbane, QLD, Australia; ^2^Extreme Environments Laboratory, Department of Sport and Exercise Science, University of PortsmouthPortsmouth, UK

**Keywords:** gastrointestinal temperature, reliability, validity, ingestible sensor, measurement error, heat, cold, thermoregulation

## Abstract

An accurate measure of core body temperature is critical for monitoring individuals, groups and teams undertaking physical activity in situations of high heat stress or prolonged cold exposure. This study examined the range in systematic bias of ingestible temperature sensors compared to a certified and traceable reference thermometer. A total of 119 ingestible temperature sensors were immersed in a circulated water bath at five water temperatures (TEMP A: 35.12 ± 0.60°C, TEMP B: 37.33 ± 0.56°C, TEMP C: 39.48 ± 0.73°C, TEMP D: 41.58 ± 0.97°C, and TEMP E: 43.47 ± 1.07°C) along with a certified traceable reference thermometer. Thirteen sensors (10.9%) demonstrated a systematic bias > ±0.1°C, of which 4 (3.3%) were > ± 0.5°C. Limits of agreement (95%) indicated that systematic bias would likely fall in the range of −0.14 to 0.26°C, highlighting that it is possible for temperatures measured between sensors to differ by more than 0.4°C. The proportion of sensors with systematic bias > ±0.1°C (10.9%) confirms that ingestible temperature sensors require correction to ensure their accuracy. An individualized linear correction achieved a mean systematic bias of 0.00°C, and limits of agreement (95%) to 0.00–0.00°C, with 100% of sensors achieving ±0.1°C accuracy. Alternatively, a generalized linear function (Corrected Temperature (°C) = 1.00375 × Sensor Temperature (°C) − 0.205549), produced as the average slope and intercept of a sub-set of 51 sensors and excluding sensors with accuracy outside ±0.5°C, reduced the systematic bias to < ±0.1°C in 98.4% of the remaining sensors (*n* = 64). In conclusion, these data show that using an uncalibrated ingestible temperature sensor may provide inaccurate data that still appears to be statistically, physiologically, and clinically meaningful. Correction of sensor temperature to a reference thermometer by linear function eliminates this systematic bias (individualized functions) or ensures systematic bias is within ±0.1°C in 98% of the sensors (generalized function).

## Introduction

The human body's capacity to regulate its internal temperature ensures optimal health and physiological function when exposed to a wide range of ambient environments (Kenney et al., 2004). In environments that are conducive to heat stress (high ambient temperature, humidity, radiant heat sources, and low air movement) increased sweating and skin blood flow facilitate thermoregulation. However, performing high intensity physical activities combined with wearing protective clothing can exacerbate and overwhelm the capacity to maintain thermal homeostasis (Montain et al., 1994). Such exposures are commonplace in sports [e.g., the American National Football League (Armstrong et al., 2010) and soccer (Taylor L. et al., 2014)], in occupational settings [e.g., fire fighters (Larsen et al., 2015; Walker et al., 2015) and other emergency first responders (Costello et al., 2015)], and in military operations (Hunt et al., 2016). Consequently, internal body temperatures can rise to dangerous extremes resulting in fatal heat stroke if the elevation in body temperature is left unchecked (Carter et al., 2005; Grundstein et al., 2012). Conversely, if individuals are exposed to extreme cold environments, particularly for a long duration of time, hypothermia becomes a serious risk to health and performance (Castellani and Tipton, 2015; Brazaitis et al., 2016). For this reason an accurate measure of core body temperature is critical to the development of safe guidelines for individuals, groups and teams undertaking physical activity in situations of prolonged exposure to extreme environments.

Ingestible temperature sensors have become a valuable and commonly used tool for monitoring core body temperature outside the clinical context (Taylor N. A. S. et al., 2014), particularly in athletic and occupational settings that require freedom of movement (Stewart and Hunt, 2011; Hunt et al., 2016). Comparisons of ingested temperature sensors with the clinical measure of rectal temperature have concluded that ingested sensors provide a valid measure of core body temperature during exercise in hot environments both indoors and outdoors (Casa et al., 2007; Ganio et al., 2009), as well as indoors in cold environments (Bagley et al., 2011). In addition to concluding that ingestible sensors are a valid surrogate of rectal temperature and oesophageal temperature, it has been recommended that the ingestible sensors are corrected to align with a certified thermometer (Byrne and Lim, 2007). To ensure optimal measurement accuracy, research laboratories are advised to assess the validity and reliability of temperature measurement devices by immersion in a sterile water bath at several temperatures in the physiological range (Simpson et al., 2006). A linear correction is then applied to align the measurements with a certified and traceable reference thermometer. Calibration of thermometry devices at regular intervals has been shown to improve accuracy of aging instruments and ensures their sensitivity and specificity is preserved for the diagnosis of various conditions and pathologies (Simpson et al., 2006).

Although ingestible temperature sensors are becoming increasingly utilized for both laboratory and field investigations of human thermoregulation, few researchers report performing calibrations. A brief review of several leading journals in the areas of exercise physiology, sports medicine, and applied ergonomics (including Frontiers in Physiology, Medicine and Science in Sports and Exercise, Journal of Applied Physiology, and Applied Ergonomics) revealed 52 papers published between 2006 and 2016 reported temperature from an ingested sensor as a primary outcome measure. However, only eight studies (14%) reported performing a calibration of the temperature sensors prior to ingestion (Byrne et al., 2006; Gant et al., 2006; Mermier et al., 2006; Goosey-Tolfrey et al., 2008; Wilkinson et al., 2008; Logan-Sprenger et al., 2012; Pyke et al., 2015; Stewart et al., 2017). The absence of a temperature correction procedure potentially introduces error into the measurement for two reasons. Firstly, it remains unclear if the manufacturers claimed level of accuracy is in fact achieved by the device, and secondly, the level of agreement between ingestible sensors cannot be quantified.

A pilot study has demonstrated the importance of conducting a correction procedure by exposing these sources of error on a limited sample of sensors (Hunt and Stewart, 2008). Manufacturer claims indicate an accuracy of ±0.1°C (HQInc.^1^ and VitalSense^2^, which coincides with the recommended accuracy for measurement of internal body temperature (Moran and Mendal, 2002), yet one out of three ingestible sensors recorded temperature outside this range (Hunt and Stewart, 2008). Furthermore, a statistically significant discrepancy (*F* = 10.818, *p* < 0.001) was reported between the sensors (Hunt and Stewart, 2008). The pilot study recommended that corrections be performed by a linear regression developed between the ingested sensor and a certified traceable thermometer in a water bath heated to a minimum of four discrete temperatures in the physiological range. However, the study was limited by a small sample size of only three sensors. Consequently, the small sample size leaves it unclear if the one sensor that recorded temperature outside the ±0.1°C standard was a simple anomaly, or a true representation of the proportion of sensors that might be expected in a larger sample. Therefore, the aims of this study were to examine the range in systematic bias in a large sample of ingestible temperatures sensors than previously reported and to evaluate the linear regression approach to temperature correction.

## Methods

A total of 119 ingestible temperature sensors (CorTemp, HQinc, Palmetto FL, USA) were evaluated as part of this investigation. All sensors where tested within their individual expiration dates. Each sensor was immersed in a circulated water bath (model 350, Contherm Scientific Inc., New Zealand) at five water temperatures that encompassed the physiological range of human body core temperature anticipated during physical activities. The average water bath conditions were: TEMP A: 35.12 ± 0.60°C, TEMP B: 37.33 ± 0.56°C, TEMP C: 39.48 ± 0.73°C, TEMP D: 41.58 ± 0.97°C, and TEMP E: 43.47 ± 1.07°C. A manufacturer calibrated and traceable thermometer (TL-1W, ThermoProbe, USA) with certified accuracy of ±0.06°C was suspended in the water bath and served as the reference measure of water temperature. Once the water temperature was considered stable (±0.05°C) for at least 5 min and the sensors had been submerged for >4 min to allow equilibration with the water temperature (Hunt and Stewart, 2008), sensor temperature was recorded at 10 s intervals and averaged over a 1 min period. The temperature variation in the water bath was <0.01°C during recording; a level of variation comparable to similar studies (Harper Smith et al., 2010).

Bland-Altman plots with 95% limits of agreement analysis, accounting for the repeated measurement conditions with each sensor, were performed to describe the agreement between the ingestible sensors and references temperatures (Bland and Altman, 2007). Repeated measures analysis of variance with pairwise comparisons was performed to evaluate any effect of water bath temperature on systematic bias, with significance accepted at α < 0.05. Sensor calibrations were performed by linear regression; Firstly as an individualized linear regression specific to each individual sensor. To examine the potential to utilize a generalized linear regression to calibrate ingestible sensors, the sensors were randomly allocated into development (*n* = 52) and verification (*n* = 67) groups, whereby the generalized linear regression was generated as the average slope and intercept of the development sensors, and applied to the verification sensors. Finally, ingestible sensors with a systematic bias > ±0.5°C were removed from the analysis (*n* = 4) and the generalized linear regression was calculated on the remaining development sensors (*n* = 51) and applied to the remaining verification sensors (*n* = 64).

## Results

**Figure 1:**
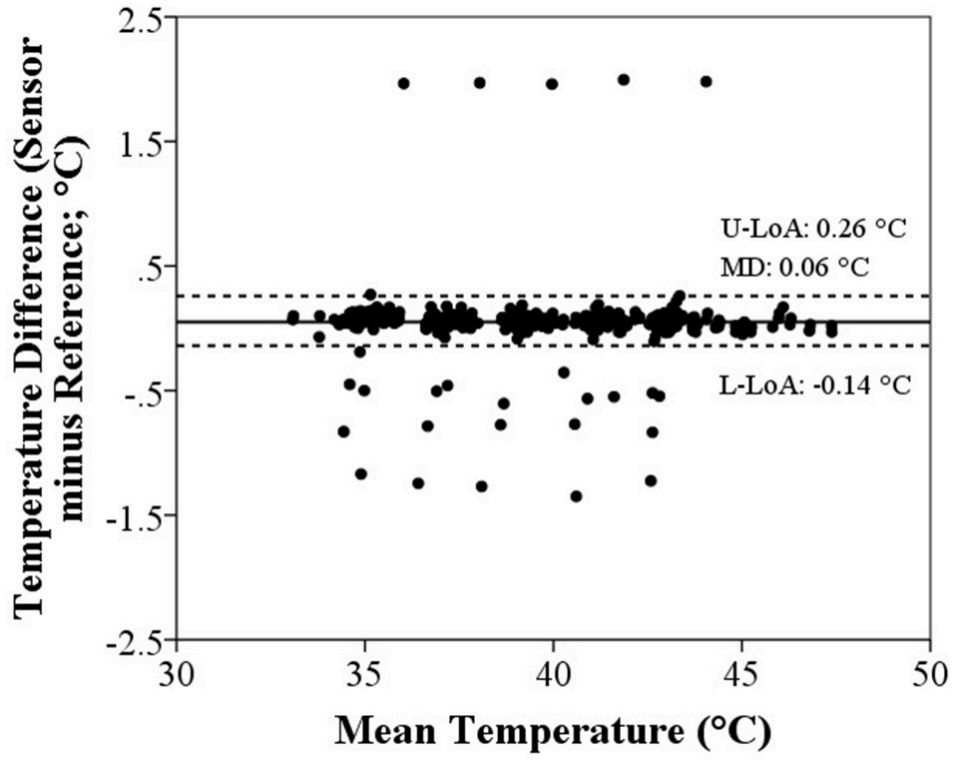
**Bland-Altman plot of the agreement between ingestible sensor and reference temperatures**. Solid and dashed lines represent the mean difference (MD) and limits agreement (LoA; upper–U, and lower–L), respectively.

A total of 71 (59.7%) sensors demonstrated a systematic bias greater than the measurement accuracy of the reference thermometer (±0.06°C). Thirteen sensors (10.9%) demonstrated a systematic bias > ±0.1°C, of which 4 (3.3%) were > ±0.5°C. Overall, sensors tended to overestimate the reference thermometer, with systematic bias averaging 0.06 ± 0.24°C (Figure 1). Limits of agreement (95%) indicated that systematic bias would likely fall in the range of −0.14 to 0.26°C, highlighting that it is possible for temperatures measured between sensors to differ by 0.40°C. However, the extremes of systematic bias of individual sensors ranged from −1.35 to 2.00°C. The systematic bias was significantly different across the range of water bath temperatures (TEMP A: 0.07 ± 0.23; TEMP B: 0.06 ± 0.24; TEMP C: 0.05 ± 0.24; TEMP D: 0.05 ± 0.24; TEMP E: 0.05 ± 0.24; *F* = 14.199, *P* < 0.001), with Temp A significantly different to all other conditions (*P* < 0.001).

A strong linear relationship was observed between the reference and sensor temperatures, with a correlation coefficients (r) of 1.00 consistently for each individual ingestible sensor. Due to the strength of the relationship it was accepted that linear regression was a suitable method for correction of the sensor temperature to the reference temperature. An individualized linear regression, specific to each ingestible sensor, corrected the mean systematic bias to 0.00°C, and limits of agreement (95%) to 0.00–0.00°C. As a result, the proportion of sensors with a systematic bias < ±0.1°C rose to 100% following correction with individual regression equations.

**Figure 2:**
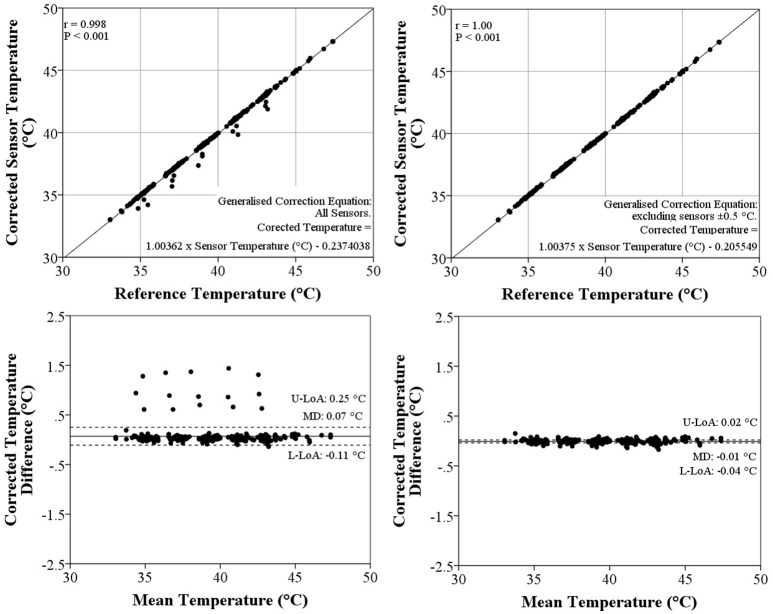
**Linear Regressions (Top) and Bland-Altman Plots (Bottom) comparing the Corrected Sensor Temperature with the Reference Temperature**. Graphs on the left report the generalized linear equation including all sensors, while graphs on the right show the generalized equation excluding sensors outside ±0.5°C of the reference thermometer. Solid and dashed lines represent the mean difference (MD) and limits agreement (LoA; upper–U, and lower–L), respectively.

The generalized linear regression generated from the group of development sensors (Corrected Temperature (°C) = 1.00362 × Sensor Temperature (°C) − 0.2374038) produced a mean systematic bias of 0.07 ± 0.21°C when applied to the verification sensors (Figure 2). The limits of agreement (95%) of the verification sensors ranged between −0.11 and 0.25 (Figure 2). With this generalized correction only 94.0% of the verification sensors showed a systematic bias of < ±0.1°C. However, when sensors with systematic bias > ±0.5°C were removed from the analysis, the generalized linear regression (Corrected Temperature (°C) = 1.00375 × Sensor Temperature (°C) − 0.205549) corrected systematic bias among the verification sensors to −0.01 ± 0.04°C, with limits of agreement (95%) of −0.04 to 0.02°C (Figure 2). Consequently the proportion of sensors with a systematic bias < ±0.1°C was 98.4%.

## Discussion

Overall, 89.1% (*n* = 106) of ingestible temperature sensors measured temperature in the physiological range within ±0.1°C. The remaining 11.7% (*n* = 13) displayed a wide range in systematic bias (Figure 1) which highlights the importance of performing a calibration procedure prior to administering the sensor for ingestion. An individual linear function for each sensor was required to eliminate the systematic bias from the reference thermometer and therefore between different sensors, however, these findings also demonstrate that an average linear function could be used to ensure error between sensors is restricted to an acceptable range. Provided that sensors with a systematic bias > ±0.5°C are identified and excluded, correcting sensor temperature readings by the following equation: corrected temperature (°C) = 1.00375 × sensor temperature (°C) − 0.205549; achieved a 98.4% success rate in meeting the ±0.1°C criteria for acceptable measurement accuracy (Figure 2).

The proportion of sensors with a systematic bias outside ±0.1°C was smaller in the present study than previously observed in an initial pilot study of only three sensors (Hunt and Stewart, 2008). The present study has evaluated a larger sample of 119 sensors allowing greater confidence in the actual proportion of inaccurate sensors being identified at ~11%. Furthermore, this study has revealed that a small yet important proportion of sensors have a much greater systematic bias than previously reported, in excess of ±0.5°C. These discrepancies have important practical implications for monitoring body core temperature in athletic and occupational settings. For instance, test re-test reliability indicates that intestinal temperature can vary by up to 0.34°C between repeat exercise trials (Ruddock et al., 2014). Therefore, detection of a meaningful difference in body core temperature during experimental trials must ensure that the combined error of ingestible sensors (which are one use only) is below this value. From the proportions observed in this study, it is evident that 3% of sensors may provide inaccurate temperature measurement (> ±0.5°C) if uncorrected to a reference thermometer. Consequently, in an experimental trial of 15 participants performing two exercise conditions for example, it would be expected that at least one participant's results would be erroneous due to measurement device error instead of a treatment effect. The literature identified that utilized the ingestible sensor technology but without performing a temperature correction (40 out of the 52 studies provided enough information to determine the number of sensors used), used an average of 28 (range: 8–96) sensors per study. Proportionately, the present findings suggest that at least one participant's data would be erroneous in each of these studies. Consequently these findings highlight the necessity of performing a pre-ingestion temperature assessment of ingestible sensors in a water bath.

Correction of sensor temperature to a reference thermometer by linear regression was confirmed as an appropriate technique in the present study. The systematic bias of sensor temperature was effectively eliminated by the application of a linear function specific to each ingestible sensor. While an individual linear function was identified as the optimal correction procedure, there is also considerable time cost to researchers and practitioners in implementing this strategy. Alternatively, an average linear function has been proposed. By this method sensor temperature could be compared to a reference thermometer at a single water bath temperature in the physiological range and, if found to be within ±0.5°C, could subsequently be corrected by the average linear function [corrected temperature (°C) = 1.00375 × sensor temperature (°C) − 0.205549)]. This technique would restrict systematic bias to the required ±0.1°C accuracy range for 98.4% of sensors.

The ingestible temperature sensors are most commonly employed for monitoring extremes of body temperature experienced in real-world situations, as such the accuracy of the temperature measurement is of paramount importance at the extremes of the physiological range. However, it has been reported that the differences between ingestible sensor and rectal temperatures tended to be greater in those who showed the highest elevations in rectal temperature (Casa et al., 2007). Similarly, in studies examining ingestible temperature sensors in a water bath, greater systematic bias has been reported as temperatures progress toward the extremes of the physiological range (Hunt and Stewart, 2008; Travers et al., 2016). In the present investigation systematic bias was significantly different at the lowest temperature condition (TEMP A: 35.12 ± 0.60°C) compared to the warmer temperature conditions. This finding further highlights the importance of ensuring accurate temperature measurement through correction before ingestion. According to the National Athletic Trainers Association Position Statement on Environmental Cold Injuries a core temperature of 35°C is an indicator of mild hypothermia (Cappaert et al., 2008). Therefore, accurate measurements of core temperature are required in the field for assessment and diagnosis of injuries associated with exposure to the environment. In addition, this finding also highlights that sensor corrections need to be based on reference temperatures measured within the physiological range. For if correction equations were to be based on temperatures outside the physiological range an incorrect linear function may be developed.

A limitation of the present study was that only one model of ingestible sensor was evaluated, yet there are several others on the commercial market. Systematic bias of other ingestible sensors such as the VitalSense and the e-Celcius, has been observed in the range of 0.18–0.34°C, respectively (Travers et al., 2016). In addition, the difference in accuracy at the extremes of the physiological range may also differ as previous evaluations have displayed sensors overestimating at low temperatures and underestimating at high temperatures (Chapon et al., 2012). Therefore, caution should be exercised before implementing the average linear function developed here to other models of ingestible sensor.

## Recommended calibration method

Based on the findings of the present study the following method is recommended for calibration of ingestible temperature sensors. Equipment requirements include a circulated and sterile water bath capable of maintaining a stable water temperature in the expected physiological range, as well as a certified and traceable reference thermometer with accuracy < ±0.1°C. Also, ensure the ingestible sensors are within the manufacturer's expiration date.

### Individual sensor calibration

To develop an individual sensor calibration equation, sensor, and reference temperatures should be measured in at least four water bath temperatures. For each water bath temperature ensure:

1. Sufficient time (>4 min) is allowed for water temperature and sensor temperature to stabilize before the measurement is recorded.
2. Repeat this procedure for each water bath temperature.
3. Plot the linear relationship between sensor and reference temperatures.
4. Utilize the individualized linear equation to correct raw data from the ingestible sensor. Raw data from the sensor can either be corrected by the linear regression in a post-processing of the data after the exercise trial is completed, or could be incorporated into the investigators viewing of the data if real-time corrections are required during a trial.

### Generalized sensor calibration

To utilize the generalized linear correction presented in this paper, sensor and reference temperature should be measured in at least one water bath temperature in the physiological range anticipated depending on the expected values for the activity to be monitored or the type of research study).

1. Sufficient time (>4 min) is allowed for water temperature and sensor temperature to stabilize before the measurement is recorded.
  - If sensor temperature is within ±0.5°C of the reference thermometer, the following correction can be applied to the raw sensor temperature data:
    $$\begin{array}{l} \text{Corrected Temperature }({}^{\circ}\text{C})=1.00375\\ \;\times \text{ Sensor Temperature }({}^{\circ}\text{C})-0.205549 \end{array}$$
  - If sensor temperature is outside ±0.5°C then an individual sensor calibration should be performed (see individual sensor calibration above).

The timing of sensor calibration prior to ingestion will be an important consideration in trial preparation and depend on the type of study being undertaken. For example, a study of body temperature during a mass participation endurance event would require all sensors to be calibrated in the same time window prior to the event. Alternatively, a laboratory based study with repeated trials over weeks or months requires the calibration procedure to be performed within a short time period before each trial. Performing the calibration procedure in the time window immediately prior to its ingestion will ensure battery life of the sensor is sufficient to record data for the duration of the specific trial activity being undertaken by the participant. If for practical limitations the trial activity necessitates a prolonged period between calibration and ingestion (such as long distance travel to a test location), some models of ingestible sensor can be deactivated (switched off) in the interim to preserve battery life (Hunt and Stewart, 2008).

## Conclusion

In conclusion, this study has confirmed that ingestible temperature sensors require correction by linear regression. The optimal technique for reducing systematic bias of the sensor is the development of an individualized linear function to correct temperature data to a reference thermometer. However, application of an average linear function [corrected temperature (°C) = 1.00375 × sensor temperature (°C) − 0.205549] in sensors with an initial accuracy within ±0.5°C of the reference thermometer, was also effective at reducing systematic bias to < ±0.1°C in 98.4% of sensors.

## Author contributions

All authors made substantial contributions to the design of this study and/or acquisition, analysis, and interpretation of the data. Additionally, AH and AB wrote the manuscript in conjunction with critical review from DB, JC, and IS. All authors approve of the final version to be published and agree to be accountable for all aspects of the work.

## Funding

This project was financially supported by the Australian Government, managed by the National Security Science & Technology Centre within the Defence Science & Technology Organization (DSTO), and the US Government through the Technical Support Working Group within the Combating Terrorism Technical Support Office (CTTSO). Financial support by DSTO and CTTSO does not constitute an express or implied endorsement of the results or conclusions of the project by DSTO, CTTSO, nor the Department of Defense.

### Conflict of interest statement

The authors declare that the research was conducted in the absence of any commercial or financial relationships that could be construed as a potential conflict of interest.

## Footnotes

^1^http://www.hqinc.net/

^2^https://bmedical.com.au/product/core-body-temperature-capsule-ingestable-jonah/
